# Longevity

**Published:** 1854-02

**Authors:** 


### LONGEVITY.

The following table shows that men have attained a good old age, and there is no reason to suppose that these may not be the average ages of men and women, if modes of life were adopted, which would involve the fundamental principles, by which these men lived thus long, the great features being TEMPERANCE AND MODERATE INDUSTRY:

| | | | |
|---|---|---|---|
| Dryden, | 70 | Sophocles, | 90 |
| Petrarch, | 70 | Livia, | 90 |
| Lesage, | 70 | Eli, | 90 |
| Linnæas, | 71 | Protagoras, | 90 |
| Locke, | 73 | Lewenhoeck, | 91 |
| La Fontaine, | 74 | Cato, | 91 |
| Rev. Dr. Wardlow, | 75 | Hans Sloane, | 93 |
| Handel, | 75 | Whiston, | 95 |
| Reaumer, | 75 | Michael Angelo, | 96 |
| Gallileo, | 78 | Titian, | 96 |
| Swift, | 78 | Isocrates, | 98 |
| Roger Bacon, | 78 | Elisha, | 100 |
| Corneille, | 78 | Hervelias, | 100 |
| Marmontel, | 79 | Fontenelle, | 100 |
| Solon, | 80 | Zeno, | 100 |
| Thucydides, | 80 | Terentia, | 103 |
| Anacreon, | 80 | Stender, | 103 |
| Juvenal, | 80 | Helen Gray, | 105 |
| Kant, | 80 | Georgias, | 107 |
| Pindar, | 80 | Thomas Garrick, | 108 |
| Young, | 80 | Democritus, | 109 |
| Willard, | 80 | Joseph, | 110 |
| Sophocles, | 80 | Joshua, | 110 |
| Plato, | 81 | A. Serush, | 111 |
| Buffon, | 81 | Mittelstedt, | 112 |
| Goethe, | 82 | H. Thauper, | 112 |
| Dr. Chas. Caldwell, | 82 | R. Glen, | 115 |
| Claude, | 82 | Moses, | 120 |
| West, | 82 | Sarah, | 127 |
| Franklin, | 84 | Ishmael, | 137 |
| Metastasio, | 84 | Effingham, | 144 |
| Herschell, | 84 | Drakenberg, | 146 |
| Anacreon, | 85 | Jacob, | 147 |
| Newton, | 85 | Thomas Parr, | 153 |
| Voltaire, | 85 | Epimenides, | 157 |
| Halley, | 86 | Henry Jenkins, | 169 |
| Simeon, | 90 | Abraham, | 175 |
| Fabius, | 90 | Isaac, | 180 |

Among the preceding names are found all the occupations of life, from the philosopher to the common day-laborer, selected from all nations, and of all ages, from the days of Abraham down to the present time, and if no nation, or age, or sex, or clime, or ordinary occupation necessarily prevents men from arriving at old age, that old age must be generally attainable, if the proper conditions are met. It is the design of this Journal to inculcate these conditions. To do it early, is of the highest importance, as it gives every advantage; hence the special desire of the Editor that parents generally should have their children, at least those above fifteen years of age, become subscribers to this periodical.

